# Induction and Enhancement of Cardiac Cell Differentiation from Mouse and Human Induced Pluripotent Stem Cells with Cyclosporin-A

**DOI:** 10.1371/journal.pone.0016734

**Published:** 2011-02-22

**Authors:** Masataka Fujiwara, Peishi Yan, Tomomi G. Otsuji, Genta Narazaki, Hideki Uosaki, Hiroyuki Fukushima, Koichiro Kuwahara, Masaki Harada, Hiroyuki Matsuda, Satoshi Matsuoka, Keisuke Okita, Kazutoshi Takahashi, Masato Nakagawa, Tadashi Ikeda, Ryuzo Sakata, Christine L. Mummery, Norio Nakatsuji, Shinya Yamanaka, Kazuwa Nakao, Jun K. Yamashita

**Affiliations:** 1 Laboratory of Stem Cell Differentiation, Stem Cell Research Center, Institute for Frontier Medical Sciences, Kyoto University, Kyoto, Japan; 2 Department of Medicine and Clinical Science, Kyoto University Graduate School of Medicine, Kyoto, Japan; 3 Department of Cardiovascular Surgery, Kyoto University Graduate School of Medicine, Kyoto, Japan; 4 Stem Cell and Drug Discovery Institute, Kyoto Research Park, Kyoto, Japan; 5 Laboratory of Embryonic Stem Cell Research, Stem Cell Research Center, Institute for Frontier Medical Sciences, Kyoto University, Kyoto, Japan; 6 Department of Cell Growth and Differentiation, Center for iPS Cell Research and Application (CiRA), Kyoto University, Kyoto, Japan; 7 Department of Physiology and Biophysics, Kyoto University Graduate School of Medicine, Kyoto, Japan; 8 Department of Reprogramming Science, Center for iPS Cell Research and Application (CiRA), Kyoto University, Kyoto, Japan; 9 Department of Anatomy and Embryology, Leiden University Medical Centre, Leiden, the Netherlands; 10 Department of Development and Differentiation, Institute for Frontier Medical Sciences, Kyoto University, Kyoto, Japan; 11 Institute for Integrated Cell-Material Sciences (iCeMS), Kyoto University, Kyoto, Japan; 12 Gladstone Institute of Cardiovascular Disease, San Francisco, California, United States of America; Clinica Universidad de Navarra, Spain

## Abstract

Induced pluripotent stem cells (iPSCs) are novel stem cells derived from adult mouse and human tissues by reprogramming. Elucidation of mechanisms and exploration of efficient methods for their differentiation to functional cardiomyocytes are essential for developing cardiac cell models and future regenerative therapies. We previously established a novel mouse embryonic stem cell (ESC) and iPSC differentiation system in which cardiovascular cells can be systematically induced from Flk1^+^ common progenitor cells, and identified highly cardiogenic progenitors as Flk1^+^/CXCR4^+^/VE-cadherin^−^ (FCV) cells. We have also reported that cyclosporin-A (CSA) drastically increases FCV progenitor and cardiomyocyte induction from mouse ESCs. Here, we combined these technologies and extended them to mouse and human iPSCs. Co-culture of purified mouse iPSC-derived Flk1^+^ cells with OP9 stroma cells induced cardiomyocyte differentiation whilst addition of CSA to Flk1^+^ cells dramatically increased both cardiomyocyte and FCV progenitor cell differentiation. Spontaneously beating colonies were obtained from human iPSCs by co-culture with END-2 visceral endoderm-like cells. Appearance of beating colonies from human iPSCs was increased approximately 4.3 times by addition of CSA at mesoderm stage. CSA-expanded human iPSC-derived cardiomyocytes showed various cardiac marker expressions, synchronized calcium transients, cardiomyocyte-like action potentials, pharmacological reactions, and ultra-structural features as cardiomyocytes. These results provide a technological basis to obtain functional cardiomyocytes from iPSCs.

## Introduction

Induced pluripotent stem cells (iPSCs) are novel pluripotent stem cells generated from adult tissues by reprogramming originally with transduction of a few defined transcription factors, such as Oct4, Sox2, Klf4, and c-myc [1], [2]. Establishment of iPSC lines from adult human tissue is facilitating development of cell transplantation-based regenerative strategies and establishment of patient-derived cells as disease models. Efficient differentiation and dissecting the differentiation mechanisms of target cells would significantly contribute to elucidate the pathophysiology of diseases and provide a platform for developing new therapeutic strategies for specific diseases through such as drug discovery [3], [4].

Cardiomyocytes are a major target of regenerative medicine. Although cardiomyocyte differentiation has been reported from various progenitor and adult cell sources (e.g. bone marrow, cardiac biopsies, adipose tissue, umbilical cord, mesenchymal cells, etc), overall, the efficiencies of functional cardiomyocyte appearance have been still variable (<1–5%) [5]. Pluripotent cells, embryonic stem cells (ESCs) and iPSCs have thus emerged as among the most promising stem cell sources for inducing functional cardiomyocytes in vitro. Several induction and purification methods have been reported, starting with either mouse or human ESCs. These include stem cell aggregation in suspension and growth as embryoid bodies (EBs), co-culture with stroma cells, serum-free culture in differentiation medium, or hypoxic culture [6], [7], [8], [9], [10], [11]. Overall, the efficiency of cardiomycyte differentiation in human ESCs [6] should be still lower than in mouse ESCs [8], [11]. In view of the similarities between iPSCs and ESCs, most cardiomyocyte induction methods from iPSCs are based on those tried and tested in ESCs. Several groups have thus reported cardiomyocyte formation from mouse iPSCs using either EBs or stroma cell co-culture [12], [13], [14]. Recently, several reports on cardiomyocyte induction from human iPSCs appeared with based on EB formation though the efficiencies are still varied [15], [16], [17], [18], [19]. Other new methods robust in human iPSCs remain to be explored and maybe of particular value for preparation of transplantation cell sources as well as dissecting the differentiation mechanisms and drug discovery.

Previously, we developed a novel ESC differentiation system that recapitulates early cardiovascular development in vivo [8], [20], [21]. Flk1 (also known as vascular endothelial growth factor (VEGF) receptor-2) is the earliest differentiation marker for endothelial cells (ECs) and blood cells, and is a marker of lateral plate mesoderm [21], [22]. We induced Flk1^+^ cells from ESCs, purified them by fluorescence-activated cell sorting (FACS), and re-cultured the purified cells. We succeeded in inducing the major cardiovascular cell types from the common Flk1^+^ progenitor cells: vascular ECs, mural cells (pericytes and vascular smooth muscle cells) [20] and cardiomyocytes [8]. When purified Flk1^+^ cells were cultured on mouse bone marrow-derived stromal cells, OP9 cells, spontaneously beating cardiomyocytes as well as ECs can be induced within 3–4 days (Flk-d3-4) even from a single cell. We, thus, demonstrated that ESC-derived Flk1^+^ cells serve as cardiovascular progenitors [8], [20], [23], which was further supported with following several mouse and human studies [9], [24], [25], [26]. We also identified a Flk1^+^/CXCR4^+^/vascular endothelial cadherin^−^ (FCV) population as highly cardiogenic progenitor cells among the progeny of Flk1^+^ mesoderm cells at the single cell level [8]. That is, in an intermediate stage of ESC differentiation between Flk1^+^ mesoderm cells and cardiomyocytes (Flk-d2), purified FCV population could efficiently give rise to cardiomyocytes from a single cell. The cardiogenic potential of FCV cells was 15–20 times higher than that of other cell populations among the Flk1^+^ cell progeny. We further confirmed FCV cells can differentiate into cardiomyocytes in vivo through cell transplantation experiments [11]. FCV cells, which are detected just 1–2 days before the cardiomyocyte appearance, are so far the nearest upstream cardiac progenitors to cardiomyocytes. This system proved amenable to induce various cardiovascular cells systematically from ESCs, explore novel differentiation methods, and dissect the differentiation processes [23], [27], [28]. Indeed, we recently succeeded in demonstrating that an immunosuppressant, cyclosporin-A (CSA) showed a novel potent effect specifically on Flk1^+^ mesoderm cells to induce a dramatic increase in FCV cardiac progenitor cells and cardiomyocytes with the use of this ESC differentiation system [11]. That is, when CSA was added to Flk1^+^ cells co-cultured on OP9 cells, appearance of FCV progenitor cells and cardiomyocytes were increased by 10–20 times.

Recently, we were able to systematically induce cardiovascular cells from mouse iPSCs in a way almost identical to that using mouse ESCs [12]. Here, we combined our technologies in ESCs and iPSCs and showed that FCV cardiac progenitors and cardiomyocytes were efficiently expanded from mouse iPSCs by CSA treatment. Moreover, we extended the CSA method to human iPSCs and showed that CSA also successfully worked in human iPSC differentiation and efficiently enhanced the appearance of spontaneously beating cells. Human iPSC-derived cardiomyocytes showed expected molecular, structural and functional features of human cardiomyocytes. We, thus, succeeded in inducing and enhancing cardiac cell differentiation from both mouse and human iPSCs.

## Methods

### Antibodies

Monoclonal antibodies (MoAbs) for murine E-cadhein (ECCD2), murine Flk1 (AVAS12) were prepared and labeled in our laboratory as described previously [8], [21], [29]. MoAb for cardiac troponin-T (cTnT) (1∶2000) was purchased from NeoMarkers (Fremont, CA). For staining human ESCs and iPSCs, another MoAb for cTnT (1∶100) was from Santa Cruz Biotechnology (Santa Cruz, CA). MoAbs for murine and human α-actinin (1∶800) was from Sigma (St Louis, Mo). MoAb of phycoerithrin (PE)-conjugated AVAS12 was purchased from eBioscience (San Diego, CA). MoAbs for biotinylated-CXCR4 was purchased from BD Pharmingen (San Diego, CA). Anti-HCN4 (1∶200) and anti-Cav3.2 (1∶200) antibodies were from Chemicon (Temecula, CA). Anti-Kir2.1 (1∶200) and anti-connexin 43 (1∶200) antibodies were from Alomone (Israel) and Invitrogen (Carlsbad, CA), respectively.

### Reagents

Cyclosporin-A (a gift from Novartis Pharma) was dissolved in Dimethyl sulfoxide (DMSO) (Nacalai Tesque, Kyoto Japan) at 30 mg/mL. Dilution of 1–3 µg/mL were made in differentiation medium at the time of use. PKH67 fluorescent dye was purchased from Sigma (St. Louis, MO).

### Mouse iPSC culture

A germline-competent mouse iPSC line, 20D-17, carrying Nanog promoter-driven GFP/IRES/puromycin resistant gene (Nanog-iPS cells), was maintained as previously described [30]. Briefly, iPSCs were maintained in Dulbecco's Modified Eagle Medium (DMEM) containing 15% FCS, non-essential amino acids, 1 mmol/L sodium pyruvate, 5.5 mmol/L 2-mercaptoethanol, 50 units/mL penicillin and 50 mg/mL streptomycin on feeder layers of mitomycin-C-treated mouse embryonic fibroblast (MEF) cells carrying stably incorporated puromycin-resistance gene. OP9 stroma cells were maintained as described [21].

### Induction of mouse cardiomyocyte differentiation

Induction of Flk1^+^ cells and sorting for Flk1^+^ cells were performed as previously described [8], [12], [20]. Briefly, mouse iPSCs were first plated on to gelatin-coated dishes and cultured for 30 min to eliminate attached feeder cells, then, non-adherent cells were collected and induced to differentiation. Mouse iPSCs were cultured at a density of 1–2.5×10^3^ cells/cm^2^ in differentiation medium (DM)(alpha minimum essential medium (GIBCO, Grand Island, NY) supplemented with 10% fetal calf serum) on type IV collagen-coated dishes (Biocoat, Beckton Dickinson) or mitomycin C-treated confluent OP9 cell sheets (MMC-OP9) for 96–108 h. Cells were collected and selected by FACS to purify Flk1^+^ cells. Flk1^+^ cells were then plated on to MMC-OP9 at a density of 1–10×10^3^ cells/cm^2^ and cultured in differentiation medium to induce cardiac differentiation. CSA (1–3 µg/mL) was added to Flk1^+^ cells on OP9 cells. Medium was replaced every 2 days.

### Flowcytometry and cell sorting

FACS for differentiating mouse iPSCs was performed as described previously [8], [12], [20]. After 96–108 h of iPSC differentiation, cultured cells were harvested and stained with allophycocyanin (APC)-conjugated AVAS12 and FITC-conjugated ECCD2. Viable Flk1^+^/E-cadherin^−^ cells, excluding propidium iodide (Sigma), were sorted by FACS AriaII (Becton Dickinson). For FACS for FCV progenitor cells, after 2 days differentiation of purified Flk1^+^ cell on PKH67-stained OP9 cells (Flk-d2), cultured cells were harvested and stained with a combination of MoAbs of PE-conjugated AVAS12 and biotinylated CXCR4 followed by addition of streptoavidin-conjugated APC, and subjected to FACS analysis. PKH-negative populations were analyzed and sorted as iPSC-derived cells. The Flk1^+^/CXCR4^+^ population (which was vascular endothelial cadherin-negative) [8] was designated “FCV cells”. For FACS for cardiomyocytes, cells were harvested after 6–8 days culture of Flk1^+^ cells on OP9 cells (Flk-d6-8). Induced cardiomyocytes were selected using tetramethyl rhodamine methyl ester (TMRM) (Invitrogen) [12], a fluorescent probe to monitor the membrane potential of mitochondria. In brief, cells were dissociated with 0.25% tryprin/EDTA, then incubated in DM with 50 nmol/L TMRM at 37°C for 15 minutes. Stained cells were washed twice and selected by FACS. TMRM-high population was considered as purified cardiomyocytes in iPSCs.

### Human iPSC culture

END-2 cells were cultured as described previously [31]. Human iPS cell lines induced with transduction of four transcription factors (Oct4, Sox2, Klf4, and c-myc), 201B6 and 201B7, and Myc-negative human iPSC lines, 253G1 and 253G4 were maintained as previously described [1], [32]. 253G1 was used as the human iPS cell representative in all experiments unless stated otherwise. Induction of cardiomyocyte differentiation from human iPSCs was performed by co-culturing clumps of undifferentiated human iPSCs on END-2 cells, essentially as described previously [31]. To study the effect of CSA on cardiomyocyte differentiation, 3 µg/mL CSA was added to the culture medium on day 0 (END2-d0) or 8 (END2-d8) after start of co-culture. The number of beating colonies on END2-d12 was scored by microscopic examination. For intracellular Ca^++^ measurement and immunostaining for cTnT and actinin, beating colonies were mechanically excised, then gently dissociated by trypsin-EDTA treatment (at 37°C, 10 min), and replated on to gelatin-coated dishes. For electrophysiological analysis, beating colonies were mechanically excised and then dissociated by trypsin-EDTA with DNAse I (at 37°C, 10–15 min), and replated on to gelatin-coated dishes.

### Immunohistochemistry

Immunostaining of murine cardiomyocytes was performed as described [8], [11], [12]. Briefly, 4% paraformaldehyde (PFA)-fixed cells were blocked by 2% skimmed milk (BD, bioscience) and incubated with 1st Abs. For immunohistochemistry, anti-mouse IgG –horse radish peroxidase (HRP) (Invitrogen) was used as 2nd Abs. For immunofluorescent staining, anti-mouse, rat and rabbit immunoglobulin conjugated with Alexa 488 or 546 were used for 2nd Abs. Nuclei were visualized with DAPI (Invitrogen). Cardiomyocyte differentiation was quantified as the fluorescent intensity of cTnT staining as described [8]. Immunostaining for human cardiomyocytes, 4% paraformaldehyde (PFA)-fixed cells were processed with 0.2% Triton X100 and 1% BSA (Sigma), and incubated with 1st Abs. Stained cells were photographed with inverted fluorescent microscopy, Eclipse TE2000-U (Nikon, Tokyo, Japan), digital camera system, AxioCam HRc (Carl Zeiss, Germany), or BIOREVO BZ-9000 (Keyence, Osaka, Japan).

### Electrophysiology

Membrane potentials of single cells within a beating colony were measured using whole-cell patch clamp electrophysiology in the current-clamp mode (Axopatch200B, Axon Instruments/Molecular Devices Corp., Union City, CA). All recordings were carried out at room temperature [8].

#### Buffer compositions

Bath solution contained (in mmol/L) 140 NaCl, 5.4 KCl, 0.33 NaH_2_PO_4_, 0.45 MgCl_2_, 1.8 CaCl_2_, and 5 HEPES (pH = 7.4 with NaOH). Pipette solution contained (in mmol/L) 110 L-Aspartic acid, 30 KCl, 5 MgATP, 0.1 NaGTP, 5 K_2_Creatine phosphate, 2 EGTA, 10 HEPES, and 10 NaOH (pH = 7.2 with KOH).

Field potential (FP) recordings of the beating colonies were performed using The MED64 multi-electrode array (MEA) system (Alpha MED Scientific Inc., Osaka, Japan) at a sampling rate of 20 kHz with low path filter of 500 Hz or high path filter of 1 Hz. All MEA measurements were performed at 37°C with heated perfusion system. The signals were recorded and processed with the Mobius software (WitXerx, US). The medium were perfused 1.7 ml/min as 37°C, and then the FPs were recorded for 5 min. Subsequently, E-4031(Calbiochem, US), isoproterenol (Proternol-L®, Kowa Pharmaceutical Company, Tokyo, Japan), or propranprol (Inderal®, AstraZeneca, Japan) was added to medium (discrete colony samples were used for each drug). Then, the FPs were measured for about 10 min.

### Intracellular Ca measurement

Human iPSCs were loaded with 4 µM Quest Fluo-8 (ABD Bioquest, Inc. Sunnyvale, CA) for 30 min. Fluo-8 fluorescence (excitation at 495±10 nm and emission at 535±20 nm) of beating colony was measured every 16 msec with a back-thinned electron multiplier CCD camera (ImagEM; Hamamatsu Photonics, Hamamatsu, Japan). Four consecutive images were averaged. Ratio (F1/F0) to an image at minimum fluorescence intensity (F0) was calculated after background subtraction. The measurements were carried out at room temperature.

### Reverse Transcription Polymerase Chain Reaction (RT-PCR)

Total RNA was isolated from various kinds of cell populations with the use of RNeasy Mini Kit (QIAGEN, Valencia, CA). cDNA was synthesized by the SuperScript III First-strand Synthesis System (Invitrogen). Polymerase chain reaction was performed with the use of KOD Plus (Toyobo, Tokyo, Japan) as described [33]. Primer sequences [34] are shown in Table S1.

### Electron microscopic study

Human iPSC-derived beating colony was replated on multi-well chamber slide (NUNC Rochester, NewYork), fixed with 2% glutaraldehyde in 0.1 mol/L phosphate buffer (pH 7.4) for 30–60 min, washed and immersed with phosphate buffered saline for overnight at 4°C, and fixed in 1% buffered osmium tetroxide. The specimens were then dehydrated through graded ethanol and embedded in epoxy resin. Ultrathin sections (90 nm), double-stained with uranyl acetate and lead citrate, were examined under electron microscopy (H-7650; Hitachi, Tokyo, Japan).

### Statistical Analysis

All data were obtained from at least three independent experiments. Statistical analysis of the data was performed using Student's t-test or ANOVA. p<0.05 was considered significant. All data are shown as mean ± S.D.

## Results

### Cardiomyocyte and cardiac progenitor expansion from mouse iPSCs by CSA

Recently, we reported that functional cardiomyocytes were induced from mouse iPSCs with our differentiation method in mouse ES cells [12]. In brief, undifferentiated mouse iPSC colonies maintained on MEFs were morphologically similar to mouse ESCs. We induced mesoderm differentiation from mouse iPSCs by culturing on type IV collagen-coated dish with DM (see Methods). Flk1^+^ mesoderm cells that appeared were selected by FACS at 4.5 days of differentiation (iPS-d4.5) and then underwent a cardiomyocyte induction protocol involving co-culture on OP9 stroma cells; spontaneously beating cardiomyocytes began to appear after 3 to 4 days of culture (Flk-d3-4). Beating cells that appeared were positive for multiple cardiomyocyte markers and had electrophysological features assessed by whole-cell patch clamp as previously reported [8], [12].

**Figure 1:**
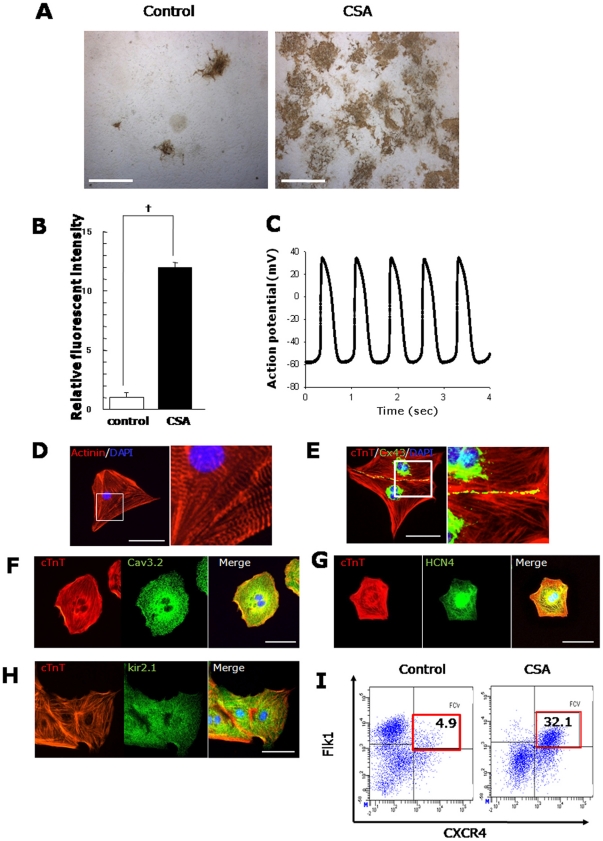
Cardiac cell expansion from mouse iPSC-derived Flk1^+^ mesoderm by CSA. **A.** Gross appearance of cardiomyocyte induction by CSA. Six days after the Flk1^+^ cell culture on OP9 cells (Flk-d6). cTnT staining (brown). Left panel: control. Right panel: CSA treatment. Scale bars = 400 µm. **B.** Quantitative evaluation of cardiomyocyte induction by fluorescent intensity of cTnT staining. Relative fluorescent intensity is indicated (n = 4, †, p<0.001 vs control). **C.** Representative action potential of iPSC-derived spontaneously beating cardiomyocytes. **D.** Sarcomeric organization in TMRM-purified cardiomyocytes at Flk-d8. Immunostaining with anti-sarcomeric α-actinin antibody (red) and DAPI (blue). Right panel shows higher magnification of boxed area. Scale bar = 25 µm. **E–H.** Double immunostaining of TMRM-purified cardiomyocytes at Flk-d8 for connexin43 (Cx43) (green) and cTnT (orange) (E), Cav3.2 (green) and cTnT (orange) (F), HCN4 (green) and cTnT (orange) (G), Kir2.1 (green) and cTnT (orange) (H). Nuclei are visualized with DAPI. Scale bars = 25 µm. I. FACS analysis for cardiac progenitor induction from mouse iPSCs by CSA. X axis: CXCR4. Y axis: Flk1. Percentages of FCV cardiac progenitor cells (double positive population; red boxes) in total Flk1^+^ cell progenies are indicated.

In the present study, we first tried to expand cardiomyocytes and cardiac progenitors from mouse iPS cells by CSA. When CSA was added to purified Flk1^+^ cells, the appearance of cTnT^+^ cardiomyocytes was increased 12-fold compared to controls (Fig. 1A, B), which was comparable with the increase observed in mouse ESCs [11]. CSA-expanded cardiomyocytes spontaneously beat and showed cardiomyocyte-like action potential (average interval: 0.74 sec, maximum diastolic potential: −58.6 mV and overshoot: 34.3 mV (n = 6)) (Fig. 1C). These cardiomyocytes also showed distinct sarcomere formation (Fig. 1D), expression of cTnT (Fig. 1E-H) and connexin 43 located at cellular boundaries (Fig. 1E). T-type calcium channel Cav3.2 (Fig. 1F), a pacemaker ion channel, HCN4 (Fig. 1G), and a ventricular ion channel, kir2.1 (Fig. 1H) were also detected in cTnT^+^ cells. We also examined the effect of CSA on the induction of FCV cardiac progenitor cells in mouse iPSCs. Addition of CSA to Flk1^+^ cells specifically increased the FCV population in mouse iPSCs to approximately 6.5 times of control. The maximum percentage of FCV cells within total Flk1^+^ cell-derived cells was more than 30% by CSA (Fig. 1I), comparable with that observed in mouse ESCs, previously [11]. CSA can thus efficiently enhance the differentiation of functional cardiomyocytes and cardiac progenitors from mouse iPSCs.

### Differentiation of cardiomyocytes from human iPSCs

**Figure 2:**
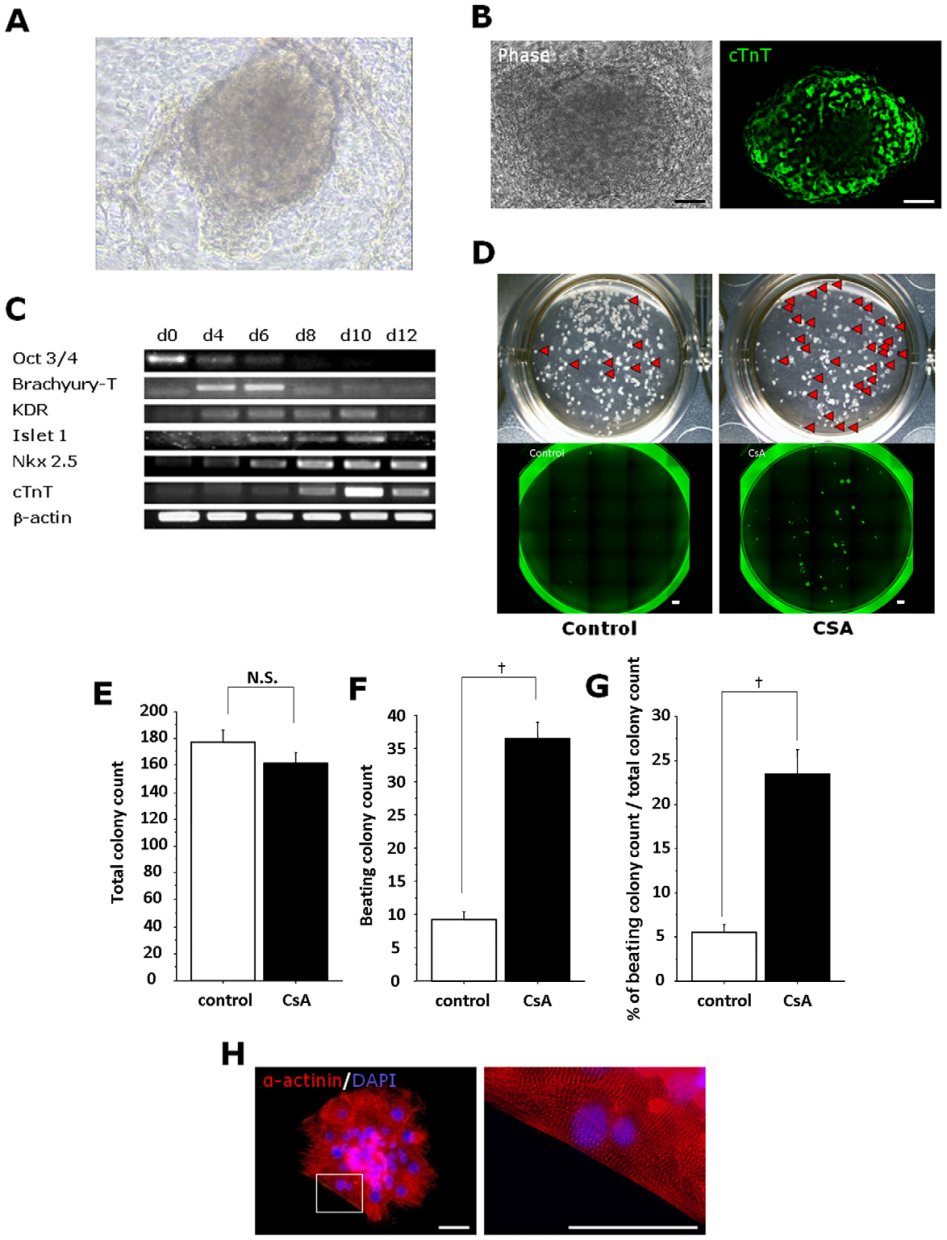
Induction and expansion of cardiomyocytes from human iPSCs. Human iPSCs were co-cultured with END-2 cells to differentiate cardiomyocytes. **A.** Gross morphology of a beating colony from human iPSCs (captured photo from Movie S1). **B.** cTnT staining of a beating colony on END-2 cells. Left panel: phase contrast image. Right panel: human cTnT staining (green). Scale bar  = 50 µm. **C.** RT-PCR analysis for differentiation markers during cardiomyocyte differentiation of human iPSCs (from END2-d0 to d12). Oct3/4: Undifferentiated cell marker, Brachyury-T: mesendoderm marker, KDR (human Flk1): mesoderm marker, Islet1: mesoderm and cardiac progenitor marker, Nkx2.5: cardiac progenitor and cardiomyocyte marker, cTnT: cardiomyocyte marker. **D.** Representative gross appearance of human iPSC-derived beating colonies at END2-d12 in 12-well dishes. Left panels: control. Right panels: CSA treatment from END2-d8. Upper panels: phase contrast images. Beating colonies are shown by red arrows. Lower panels: cTnT staining (green). **E–G** Quantitative evaluation of beating colony appearance. **E.** Total colony count (control; 177±9.7/well (12-well dishes)(n = 8), CSA; 162±8.0/well (n = 9); N.S., p = 0.237), **F.** Beating colony count (control; 9.1±1.2/well (12-well dishes)(n = 8), CSA; 36.4±2.5/well (n = 9); †, p<0.0001), and **G.** Percentages of beating colonies (control; 5.4±0.9% (n = 8), CSA; 23.5±2.8% (n = 9); †, p<0.0001) in total colonies that appeared at END2-d12. **H.** Immunostaining of actinin (red) and DAPI (blue) in dissociated cardiomyocyte colonies. The same colony is shown in Movie S2. Right panel shows higher magnification of boxed area. Sarcomere structures are evident. Scale bar = 50 µm.

We next examined cardiomyocyte differentiation from human iPSCs. We employed a human ESC differentiation method for cardiomyocytes using END-2 visceral endodermal stroma cells [31]. When human iPSCs were cultured on END-2 cells, spontaneously beating cardiomyocytes were successfully induced (Fig. 2A, Movie S1). Beating colonies were first detected after END2-d10 and became maximally evident after END2-d12. These beating colonies were positive for cTnT (Fig. 2B). During the differentiation of human iPSCs on END2 cells, sequential expression of various marker genes expected for cardiomyogenesis was observed (ex: Oct3/4; undifferentiated iPSCs, Brachyury; mesendoderm, KDR; mesoderm, islet1; mesoderm and cardiac progenitors, nkx2.5; cardiac progenitors and cardiomyocytes, cTnT; cardiomyocytes) (Fig. 2C). In our another previous study on human ESC differentiation, a Flk1 (in human, VEGF receptor-2)^+^/TRA1-60^−^ mesoderm population appeared in culture approximately 8 days after induction of differentiation [35]. When CSA was added to differentiating human iPSCs at the mesoderm stage (i.e. on END2-d8), the appearance of beating colonies was increased (Fig. 2D) although no effect was observed with the CSA treatment on undifferentiated human iPSCs (i.e. from END2-d0) (data not shown). Whereas the total number of iPSC-derived colonies that appeared was not changed (Fig. 2E), the number and percentage of beating colonies that appeared at END2-d12 were significantly increased approximately 4.0 and 4.3 times by CSA treatment, respectively (Fig. 2F, G). Approximately 23±2.7% of total colonies was beating in average, and in an optimized condition, 39% of total colonies included beating cardiomyocytes. CSA-expanded colonies maintained self-beating after a mechanical isolation and re-plating, and were positive for α-actinin with distinct sarcomere formation (Fig. 2H, Movie S2). Thus, cardiomyocyte induction from human iPSCs could be similarly enhanced by CSA. The mesoderm stage-specific effect of CSA in human iPSCs suggests the similar machinery in mouse ES/iPSCs are robustly working in human iPSC differentiation to cardiomyocytes.

### Functional features of expanded human iPSC-derived cardiomyocytes

**Figure 3:**
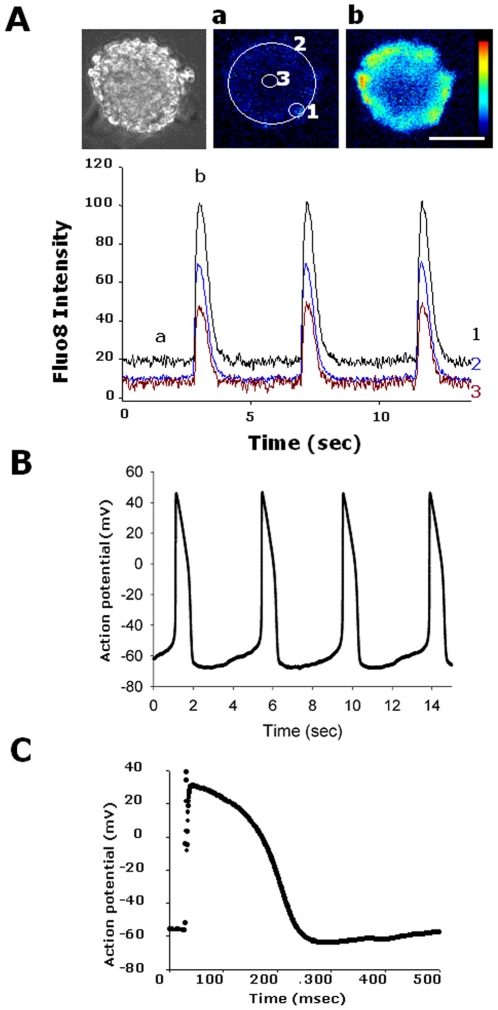
Functional analysis of expanded human cardiomyocytes. **A.** Ca^++^ transient in dissociated beating colonies. Cytoplasmic Ca^++^ change was monitored with fluo-8. Left panel: a transmission image of fluo-8 loaded iPSC colony. Middle and right panels: Fluo-8 images at the end (a) and the peak (b) of the fluorescence change. Scale bar = 50 µm. Lower panel: Time course of fluo-8 intensity change. The intensity was measured at the periphery (1), the entire colony (2) and the center (3) (ROIs shown in middle panel). Ratios (F1/F0) of the intensity to the one at the beginning of recording (F0) are indicated. Note that Ca transient is well synchronized within the colony. Real time video is shown in Movie S3. **B.** Representative action potential recorded from a cell in a beating colony. **C.** Representative single whole cell patch-cramp recording of a non-self beating human iPSC-derived cardiomyocyte after electrical stimulation.

We next evaluated functional features of CSA-expanded human iPSC-derived cardiomyocytes. Fluo-8 imaging revealed synchronized increases in intracellular Ca^++^ in beating colonies with contraction (Fig. 3A, Movie S3). Action potentials recorded by patch clamp electrophysiology identified cells with pacemaker potential (average of the interval: 4.26 sec, maximum diastolic potential: −67.6 mV overshoot: 46.6 mV (n = 6))(Fig. 3B). Replated colonies continued beating spontaneously for more than 10 months. Some isolated single cells obtained from beating colonies at 3 months culture period lost automaticity and showed some features of human ventricular cells such as action potential with rapid depolarization and prolonged plateau after electrical stimulation (Fig. 3C). These results indicate that various functional human cardiomyocytes could be induced in this system.

**Figure 4:**
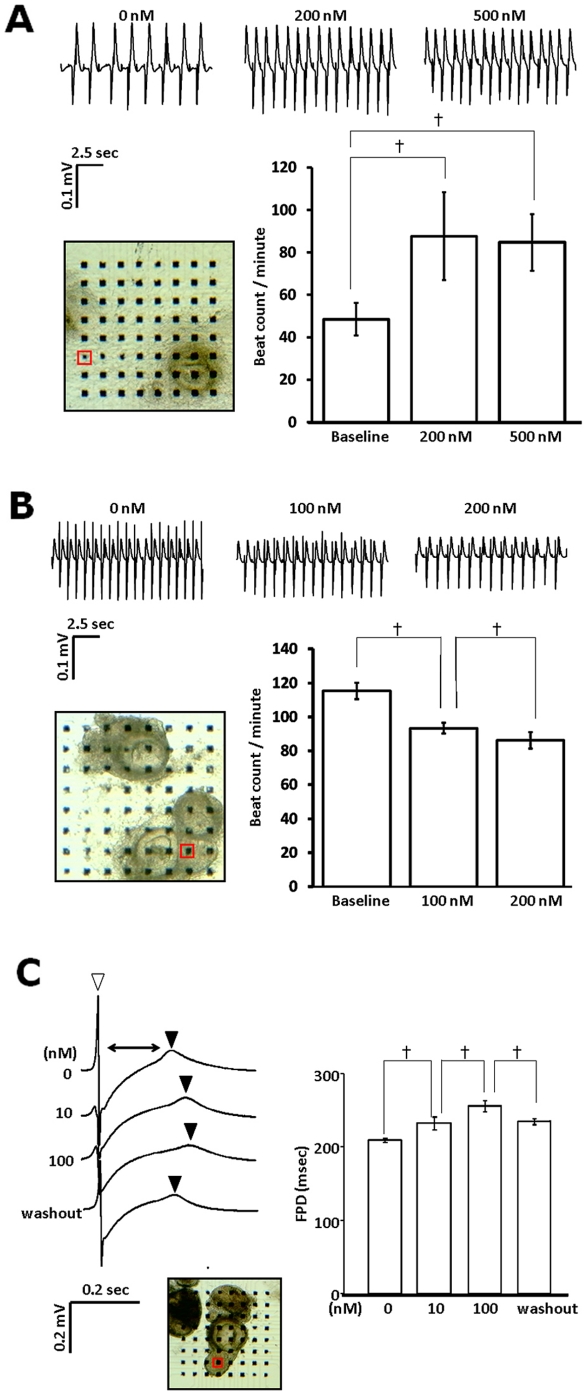
Pharmacological responses of human iPSC-derived cardiomyocytes. Field potential recordings of replated beating colonies after stimulation with isoproterenol (**A**), propranorol (**B**), and E-4031 (**C**). Photos; array of multi-electrode and replated colonies. Data recorded at electrodes in red squares are shown. **A, B.** Beating frequency (beating/minite). **C.** QT elongation. The time period from the first negative peak (open triangle) to the first positive peaks (closed triangles) reflects QT time in electrocardiogram. n = 3, †, p<0.001.

We further examined pharmacological reactions of CSA-expanded human cardiomyocytes to show the relevance as cardiac cell models. We recorded field potential of re-plated beating colonies with multi-electrode array under simulation of a β-stimulant, isoproterenol, a β-blocker, propranorol, and a HERG channel inhibitor, E-4031. Addition of isoproterenol significantly increased the beating frequency (Fig. 4A), on the other hand, propranorol significantly decreased the beating frequency (Fig. 4B). E-4031 dose-dependently prolonged the length of time from the first negative peak to first positive peak, which is corresponding to QT time in electrocardiogram (Fig. 4C). These results indicate that CSA-expanded human iPSC-derived cardiomyocytes can suffice multiple functional features as human cardiomyocyte cell models.

### Ultra structural features of expanded human iPSC-derived cardiomyocytes

**Figure 5:**
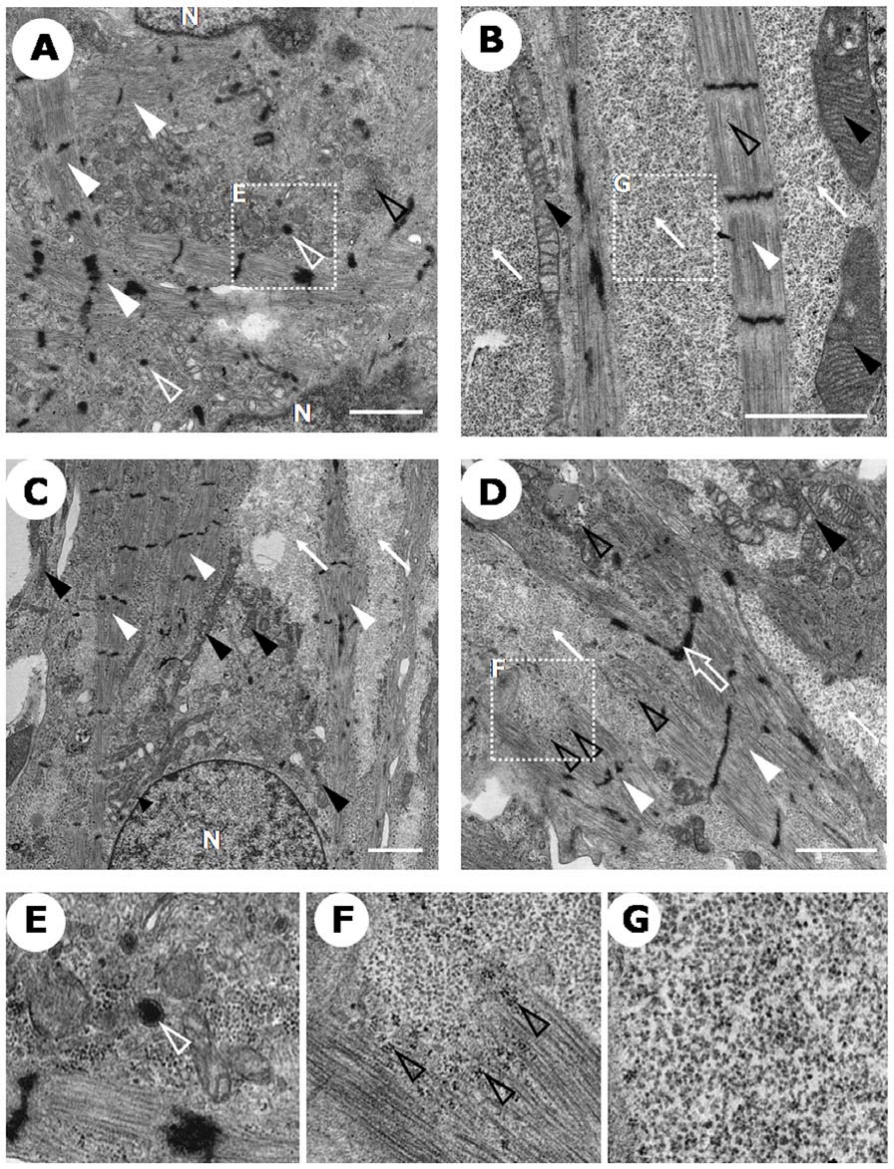
Ultrastructural analysis of human iPSC-derived cardiomyocytes. Transmission electron microscopic images of beating colonies. Myofibrils with Z-bands (white closed arrowheads in **A–D.**), mitochondria (black closed arrowheads in **B–D.**), intercalated disk-like structure with desmosome (white open arrow in **D.**), atrial sercetory granules (electron-dense granules surrounded by double membranes. White open arrowheads in **A.** and **E.** (magnified image of **A.**)), glycogen granules (electron-dense small granules. Black open arrowheads in **D.** and **F.** (magnified image of **D.**)), ribosomal granules (electron-lucent small granules. White arrows in **B–D.** and **G.** (magnified image of **B.**)). N: nucleus. Scale bar  = 2 µm, direct magnify, ×3000 (A), ×7000 (B), ×4000 (C), ×5000 (D).

We finally confirmed features of CSA-expanded human iPSC-derived cardiomyocytes at the ultrastructural level using electron microscopy. Beating colonies induced from human iPSCs resembled native cardiomyocytes, showing myofibrillar bundles with transverse Z-bands and enriched mitochondria (Fig. 5A-D). Other cardiomyocyte-specific structures, such as intercalated disks with desmosomes (Fig. 5D), atrial secretory granule-like structures (Fig. 5E), and glycogen granules (Fig. 5F) were also observed.

Together, these results indicate that *bona fide* human cardiomyocytes can be successfully induced and expanded from human iPSCs with this method.

## Discussion

Here we demonstrated the induction and expansion of cardiac progenitors and functional cardiomyocytes from iPSCs using potent and specific effect of CSA. Human cardiomyocytes with multiple expected structural and functional features could be induced with this method. This method provides a critical technological basis to obtain cardiac cells from human iPSCs.

We have demonstrated previously that CSA treatment is most effective in inducing FCV cardiac progenitor cells, the nearest upstream of cardiomyocytes in mouse ESCs [11]. Here we showed that CSA effects on FCV cardiac progenitor and cardiomyocyte induction were also completely reproduced in mouse iPSCs. Moreover, CSA also showed significant enhancing effects of cardiomyocyte differentiation from human iPSCs in the END-2 system. This is the first report to show the effect of CSA in human stem cells. In this study, we examined four human iPSC clones, 201B6, B7 (induced with four factors) [1], 253G1 and G4 (induced without c-myc) [32]. Though the basal efficiency of cardiomyocyte differentiation from 201B6, B7 and 253G4 were lower than that from 253G1, CSA treatment significantly enhanced cardiomyocyte appearance similarly in all these human iPSC clones (Figure S1). Thus, CSA robustly induced cardiogenic differentiation in mouse ESCs, iPSCs and human iPSCs regardless their species and derivation methods.

The molecular mechanisms conducting this potent CSA effect on cardiac lineage is important, but still it is unknown. Though we examined another calcineurin inhibitor, FK506, and a NF-AT inhibitor, 11R-VIVIT, both of them did not reproduce the effect of CSA [11], indicating that the cardiogenic CSA effect is mediated by other molecular target than immunosuppressing effect of CSA. Further elucidation of molecular mechanisms of CSA in cardiomyocyte differentiation would be critical for the exploration of cardiomyocyte differentiation and regeneration strategies.

CSA-expanded cardiomyocytes from human iPSCs exhibited many features sufficing as functional cardiomyocytes. Cardiomyocytes with pacemaker-like or ventricular-like action potentials were successfully induced. Nevertheless, they were still immature compared with mature adult cardiomyocytes [36], [37] and they also displayed some structural features of fetal cardiomyocytes, such as relatively low global electron density, sparse myofibrils, and abundant ribosome granules (Fig. 5). Methods for further maturation as well as specific induction and purification of the various cardiac cell types (pacemaker, atrial, ventricular, conduction system cells etc.) should be explored in future study.

Interestingly, a recent clinical report showed that CSA prevented cardiac reperfusion injury by protecting cardiomyocytes from apoptosis [38]. Cardiogenic effects of CSA in later stages of differentiation of human iPSCs imply that CSA may positively affect on endogenous cardiac progenitors to induce cardiac regeneration in patients. Though it is still unknown whether endogenous cardiac regeneration can be induced by CSA administration, our study may offer a scientific basis to support a clinical opportunity for CSA as a cardiac regenerative drug.

This novel cardiac cell differentiation method for iPSCs would thus broadly contribute to cardiac regenerative medicine by providing various options for cell preparation, transplantation strategies, and drug discovery.

## Supporting Information

Figure S1 **Quantitative evaluation of beating colony appearance in iPSC clones.**
201B6 cells: Total colony count (control; 203±6.4/well (12-well dishes)(n = 3), CSA; 193±4.0/well (n = 3); N.S., p = 0.0915), beating colony count (control; 4.0±1.0/well (n = 3), CSA; 13.7±3.5/well (n = 3); *, p<0.05), percentages of beating colonies (control; 2.0±0.5% (n = 3), CSA; 7.1±1.7% (n = 3); **, p<0.01) in total colonies that appeared at END2-d12. 201B7 cells: Total colony count (control; 204±8.3/well (n = 3), CSA; 200±2.0/well (n = 3); N.S., p = 0.43), beating colony count (control; 5.0±1.0/well (n = 3), CSA; 18.3±3.1/well (n = 3); **, p<0.01), percentages of beating colonies (control; 2.5±0.6% (n = 3), CSA; 9.2±1.5% (n = 3); **, p<0.01). 253G4 cells: Total colony count (control; 201±4.0/well (n = 3), CSA; 201±3.8/well (n = 3); N.S., p = 0.9216), beating colony count (control; 4.7±0.6/well (n = 3), CSA; 15.0±1.0/well (n = 3); **, p<0.05), percentages of beating colonies (control; 2.3±0.3% (n = 3), CSA; 7.5±0.6% (n = 3); †, p<0.001)
(TIF)

Movie S1 **A beating colony induced from human iPS cells at END2-d12 (** **Fig. 2A** **).**
(MOV)

Movie S2 **A dissociated beating colony induced from human iPS cells on END-2 cells (** **Fig. 2H** **).**
(MOV)

Movie S3 **Real time monitoring of Ca^++^ transient by Fluo-8 in dissociated beating colony induced from human iPS cells (** **Fig. 3A** **).** Clear and synchronized Ca^++^ transient is observed.
(MOV)

Table S1 **Primers for PCR.**
(RTF)
