# Supplementary material for: Induction and Enhancement of Cardiac Cell Differentiation from Mouse and Human Induced Pluripotent Stem Cells with Cyclosporin-A
Source: PLoS One. 2011 Feb 22;6(2):e16734. doi: 10.1371/journal.pone.0016734 (PMC3043062; doi:10.1371/journal.pone.0016734)
Supplement: Table S1 — Primers for PCR. (RTF) [file pone.0016734.s005.rtf]

Supplementary Table1 (Table S1)

Primers for PCR
GENEs	FORWARD PRIMERs	REVERSE PRIMERs	
OCT3/4	aac ctg gag ttt gtg cca ggg ttt	tga act tca cct tcc ctc caa cca	
T (Brachyury)	tgt ccc agg tgg ctt aca gat gaa	ggt gtg cca aag ttg cca ata cac	
KDR	cct cta ctc cag taa acc tga ttg gg	tgt tcc cag cat ttc aca cta tgg	
Isl1	cac aag cgt ctc ggg att gtg ttt	agt ggc aag tct tcc gac aa	
Nkx2.5	gcg att atg cag cgt gca atg agt	aac ata aat acg ggt ggg tgc gtg	
cTnT	ttc acc aaa gat ctg ctc ctc gct	tta tta ctg gtg tgg agt ggg tgt gg	
b-Actin	ttt gaa tga tga gcc ttc gtc ccc	ggt ctc aag tca gtg tac agg taa gc	
